# Late complications of cochlear implant: a case report of necrotizing meningoencephalitis similar to a CPA tumor

**DOI:** 10.1007/s00405-023-07956-4

**Published:** 2023-04-05

**Authors:** Vittoria Di Rubbo, Luca Morelli, Adriano Zangrandi, Lorenzo Lauda, Gianluca Piras, Mario Sanna

**Affiliations:** 1 Department of Otology and Skull Base Surgery, Gruppo Otologico, Piacenza-Rome, Italy; 2 Department of Oncohematology, Pathological Anatomy, “Guglielmo da Saliceto” Hospital, Piacenza, Italy; 3 Audiology Unit, Department of Specialistic Surgical Sciences, Fondazione IRCCS Cà Granda, Ospedale Maggiore Policlinico, Via Sforza, 35, 20122 Milan, Italy

**Keywords:** Meningoencephalitis, Cochlear implant, Late complication

## Abstract

**Objective:**

Report a case of localized necrotizing meningoencephalitis as the cause of functional hearing loss after cochlear implant (CI) surgery.

**Case report:**

A 12-year-old with bilateral CI presented to our quaternary center due to severe functional hearing loss after 11 years since left ear CI surgery. CT with contrast was conducted showing a CPA tumor-like mass. Pre-operative computed tomography (CT) scans and magnetic resonance imaging (MRI) performed at the age of 1 year showed no inner ear abnormalities and in particular no evidence of a tumor in the cerebellopontine angle (CPA).

**Conclusion:**

Following removal of the CI and the mass, histopathological, immunohistochemical and cultural examinations revealed a necrotizing meningoencephalitis, with the CI electrode as the focus.

## Introduction

Otogenic meningitis and meningoencephalitis are rare post-surgical complication which can occur following cochlear implantation [1]; predisposing conditions may include young age, otitis media, immunodeficiency, or surgical technique. These complication lead, clearly, to considerable morbidity and potential loss of hearing benefit previously achieved with the implant.

The CI, as a foreign body, may be the focus for chronic infection with a higher risk of subsequent episodes of meningitis compared to general population [2]. A biofilm formation [3] and extracellular polymeric secretions on the device surface render bacteria relatively invulnerable to the host immune response and antibiotic therapy [4, 5].

In addition, it is important to consider that infection can spread via the cochlear aqueduct intracranially causing a failure of conservative therapy such as surgical drainage and requiring a device removal [6]. The most common pathogens involved are *Streptococcus pneumoniae*, *Haemophilus influenzae*, *Pseudomonas aeruginosa, Enterococcus, Escherichia coli and Streptococcus viridians*. We present a case of a young CI recipient with localized necrotizing meningitis, similar to a CPA tumor, as a late complication.

## Case report

In April 2021, a 12-year-old boy presented to our quaternary center with a history of prelingual deafness for which he had undergone CI surgery on the left side at the age of 1 year and on the right side at the age of 3 years, both performed in another Department. Before CI surgery, the patient had completed pneumococcal and haemophilus vaccination. In addition, the patient had a history of recent right otitis with the presence of perforation of the right tympanic membrane on otoscopy. From February 2021, the patient had no longer benefited from using the left CI. The loss of benefit was rapidly progressive in the arch of a month. Particularly, hearing thresholds on both sides separately were evaluated in February 2021 in another center with a weighted four-frequency average [PTA (0.5 kHz + 1 kHz + 2 kHz + 4 kHz)/4] on pure tone audiometry in free field and speech audiometry in free field (Speech Discrimination Score, SDS): left side results were none, although the external CI processor was working, leading to the suspect of a CI internal failure. No other symptoms were reported.

**Fig. 1:**
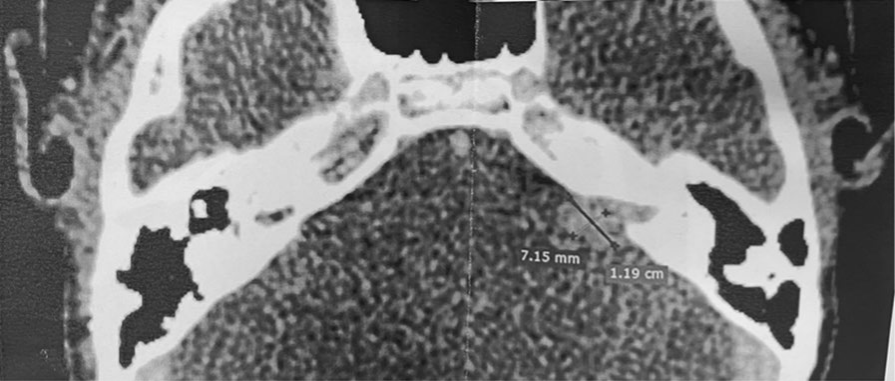
Axial contrast CT images. A contrast-enhancing mass is clear in the left CPA and IAC

**Fig. 2:**
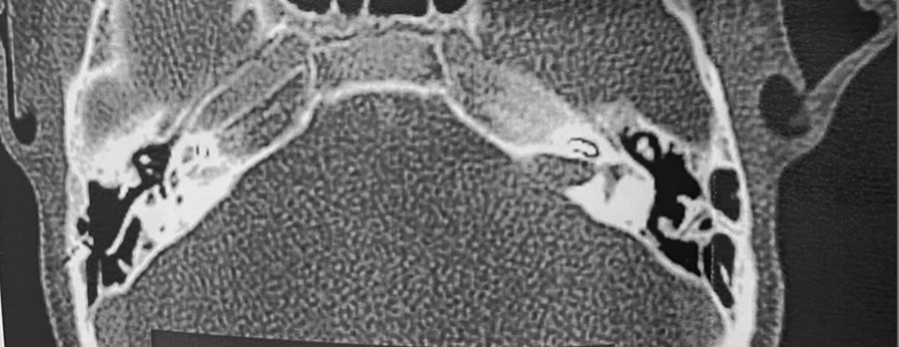
Axial contrast CT images. CI in the left basal gyrus of the cochlea and the CPA mass

**Fig. 3:**
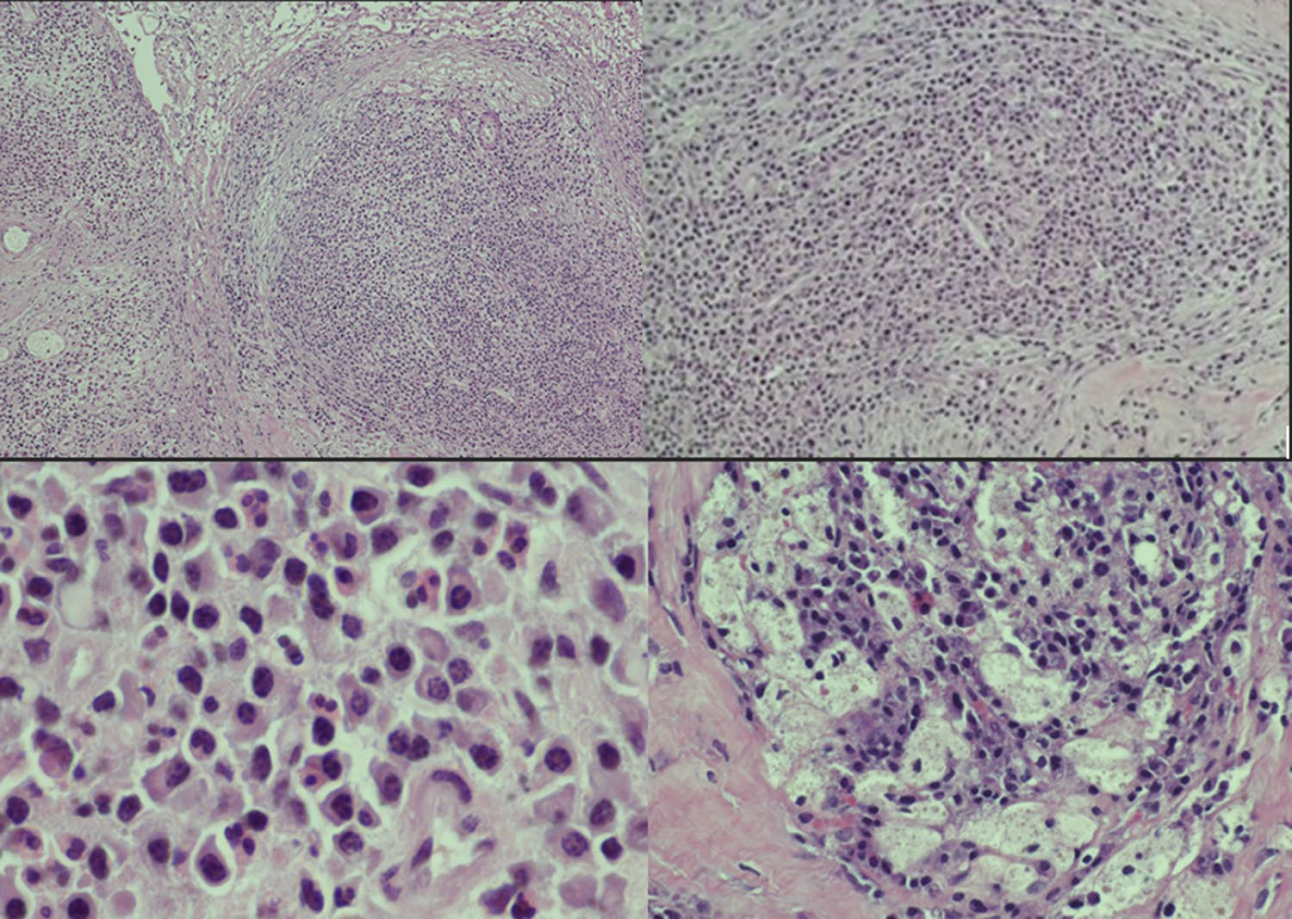
Hematoxylin–eosin. The histologic specimen exhibits a dense inflammatory infiltration admixed with vascular and fibroblastic reaction (**A**–**B**). Plasma cells and neutrophilic granulocytes are prominent (**C**) with focal macrophagic foam cell component (**D**)

In March 2021, a CT scan with contrast showed a tumor-like lesion of 7.15 mm × 11.9 mm in size in the left CPA and internal auditory canal (IAC) (Figs. 1, 2). An acoustic neuroma was suspected according to the neuroradiologists. In June 2021, the lesion was subtotally removed using a trans-labyrinthine approach and the left CI explanted; 1–2 mm of tumor was left on the brainstem due to absence of a cleavage plane. During the surgery an ossification on the array and an arachnoidal reaction with strong adhesions between the lesion and the cerebellum were observed. The lesion completely infiltrated the facial nerve and cochlear bone with the presence of polypoid tissue. Therefore, it was not possible to preserve the anatomical integrity of the facial nerve (FN). A frozen section resulted in inflammatory tissue. Microbiological examination of any part of explanted CI was assessed. Final histological examination revealed inflammatory tissue with plasma cells, granulocytes and macrophages (Fig. 3) and microbiological test showed an infection caused by *Pseudomonas aeruginosa*. A right myringoplasty was performed in July 2021. In September 2021, a cross face anastomosis was performed due to left FN paralysis. The patient showed an improvement of FN function which is currently grade IV HB [7].

## Discussion

This report demonstrates the possibility in some cases of a necrotizing meningoencephalitis as a late complication after CI surgery similar to a tumor in the CPA. Two other cases of necrotizing meningoencephalitis are described in the literature [1, 8]. It is, therefore, necessary in such cases to investigate all the risk factors that may have contributed to this condition.

One of the conditions, predisposing to infection is CI design. Older devices had higher infection rate compared with newer models. Devices with deep and narrow recesses and steep sides are more prone to bacterial attachment and biofilm [9]. Celerier [10] found biofilm staining either on the magnet, on the silicone pocket, at the emergence of the electrode array or on the extra-cochlear electrode plate. Alongside it is important to consider that an electrode positioner leads to increased rate of meningitis [11] and that infections may occur at any stage of surgery.

Staphylococcus cause most infections not only in a CI but in surgical implants in general [12]. The bacteria may be introduced as skin contaminant at the time of surgery with subsequent colonization of the implant. Staphylococcus and Pseudomonas are known to be able to develop biofilms in the presence of a foreign material [13]. The absence of microcirculation at the surface of foreign bodies leads to an insufficient host defence and delivery of antibiotics, making eradication of infection very difficult without explantation.

Local risk factors before implantation play an important role. Cunningham at all [6]. identified a history of ear disease in 52% of cases. Luntz et al. [14] found that patients who are preoperatively susceptible to otitis media also have more episodes of infections postoperatively. Good control of otitis media before implantation reduces risk of subsequent meningitis [15].

In our experience, if recurrent otitis media occurs with no benefit with the standard therapies, a subtotal petrosectomy should be considered [16, 17]. Intravenous antibiotics should be administered within 1 h before implant surgery [18]. Intraoperatively, a meticulous sterile technique must be used during the whole procedure including change of gloves immediately before handling the CI and, of course, any ear infection in implant users must be treated immediately.

## Conclusion

Severe infectious complications in CI users can occur years after implantation. If a CT scans with contrast highlights a tumor-like lesion in the cerebellopontine angle, device explantation is recommended and localized necrotizing meningoencephalitis should be considered. Nowadays many CIs are MRI compatible representing another valuable option in order to assess correctly the origin of a lesion. Microbiology examinations, including multiple swabs of any part of CI can guide in the selection of antibiotic therapy. Negative culture does not mean absence of infection; in this case, radiological follow-up with MRI with contrast can help in the discrimination between a CPA tumor and a late complication of CI.
